# Nursing care and outcome in surgical patients – why do we have to care?

**DOI:** 10.1515/iss-2019-0010

**Published:** 2019-07-25

**Authors:** Nadja Nestler

**Affiliations:** Institute of Nursing Science and Practice, Paracelsus Medical University, Strubergasse 21, 5020 Salzburg, Austria

**Keywords:** nursing competencies, nursing skill-mix, patient outcome, surgical patients

## Abstract

Nurses have an important role in patient care. They continuously work in very close contact with patients and foster the realization of activities of daily living as well as ensure quality medical treatment. For both, a high educational level is needed. A large proportion of patients with complex health situations involving chronic illnesses and multimorbidities are treated in hospitals with shortened hospital lengths of stay, changing the caring needs and the demands on nursing. Nurses must handle complex nursing tasks for which a higher educational level is indispensable, including the ability to implement evidence-based practice. In addition, studies show a correlation between the educational level of nursing staff and the health outcomes of patients. If there are too few highly educated nurses, there is an increase in patient mortality as well as the risk of patient complications, such as falls. Also, a low number of nursing staff and a high proportion of admissions decrease the quality of nursing and result in unfavorable patient outcomes. Both developments call for the necessity of a changing nursing practice and the possibilities to transform interprofessional work.

## Introduction

Good patient outcome has become exceedingly important and the primary treatment goal of hospitals. To achieve this, a well-coordinated treatment process by all participating occupational groups is needed. In addition to surgeons and other therapeutic professionals, nurses have a pivotal role. They continuously work in very close contact with patients [1]. In many situations, nurses are the first contact for patients and face not only the illness-related complications that caused the hospitalization of the patient in the first place, but also all other issues the patients experience during their hospital stay. This requires advanced professional nursing qualification and expertise in the field.

## Nursing in Germany

In Germany, nursing is an independent health profession [2]. The members of this profession carry out “their activities independently and under their own responsibility, in accordance to the level of knowledge” ([2], p. 378). Nursing is an integral part of health care and encompasses the assessment, planning, execution, and evaluation of nursing procedures [3].

Thereby, nursing includes the protection of health, the prevention of illness, and the care of physically and mentally ill and disabled persons of all ages, in all health-care and community settings [4]. Nurses play a key role in the successful delivery of health services.

Nurses take care of the outcomes of diseases and suffering. They assist patients in the accomplishment of the activities of daily living, which are normally limited or impossible after surgery. Appropriate high-level nursing competencies are necessary, because even simple tasks, such as personal hygiene or mobility, cannot be performed by the patient alone due to limitations caused by the surgery. This includes surgery-related limitations in mobility as well as in combination with other existing restrictions. Furthermore, medical treatments, such as wound care and drug therapy, also require high competencies in nursing. Both the support in activities of daily living and the assumed tasks of medical treatment ask for high-level knowledge and skills as well as competencies to act adequately.

Nurses contribute to and partake in medical diagnostics and therapy. Traditionally, these were delegated tasks by physicians, including drug administration or wound care. However, these activities are increasingly being allocated to the scope of nursing [5]. While drug administration, such as analgesic therapy, is carried out within defined therapy schemes, wound care is often carried out independently by nurses. In addition to this, nurses have become specialists in the field of pain management. For example, since the national Expert Standard for Pain Management in Nursing was published in 2005, 14,000 nurses have been trained in pain management [6]. These are called pain nurses and are pain specialists who address acute pain or are responsible for the pain management at their ward [7]. They also develop concepts for pain therapy in collaboration with physicians and evaluate its realization as well as effectiveness. Nursing specialists, like pain or wound care nurses, secure the nursing routine by directly caring for patients with complex care needs and act as consultants for colleagues and patients due to their special knowledge and skills.

However, especially against the backdrop of a changing society and the associated changing demands on the occupational field of nursing, the spectrum of tasks and the field of action in nursing are changing [5]. In Germany, the population is aging, bringing about an increase of chronic and age-associated diseases resulting in consistently higher and complex needs with increasing population numbers [8].

Chronically ill and predominantly older people tend to increasingly stay in hospitals, changing the demands on the culture of nursing [9]. In addition to treating the main acute problem, these chronically ill patients present with a number of pre-existing medical conditions that not only make them experts in their own illness in their specific life situation [10] but also make them want to participate in the treatment process, each according to their own competencies. This complicates the care process in the hospital. The restrictions associated with pre-existing diseases also affect the recovery process of the acute disease and can have a direct influence on nursing [9].

Further, there is a reduction in hospital length of stay. Since 2000, the hospital length of stay has decreased from 9.2 to 7.3 days [11], meaning that hospitalized patients are discharged earlier to return home or be transferred to institutions providing further care. Because of this shortening of hospital length of stay, there are more patients with high-level and complex nursing care needs, resulting in the need to change nursing competencies.

## Dependence of patient outcomes on care

In surgery departments, nursing care frequently occurs independently and in coordination with the treating physicians and the corresponding prescription. Usually, nurses are the primary contact for patients. They have to assess the competencies in the activities of daily living as well as after surgery and evaluate further need for assistance, and are the first ones who communicate with the physician and other occupational groups for planning the treatment process.

They support patients during all recovery phases. During the different stages of surgery (preoperative, intraoperative, and postoperative phases of surgery), diverse roles of nurses and care competencies are needed [12]. In every phase, nurses must know the entire perioperative process to adequately plan the complete treatment right from the beginning. While in the preoperative phase, nurses should prepare patients by anticipating possible fears and support the patient in preparing him or her for the surgery. In the postoperative phase, it is the aim to minimize potential complications, promote independence, and educate patients for a faster and safe recovery [13]. Prior to the surgery, nurses are expected to prepare the patients to be in the best condition for surgery [14]. Because of this, their teaching and information role becomes more important. As the patient’s length of stay is often rather short, structured pathways become more significant.

**Figure 1:**
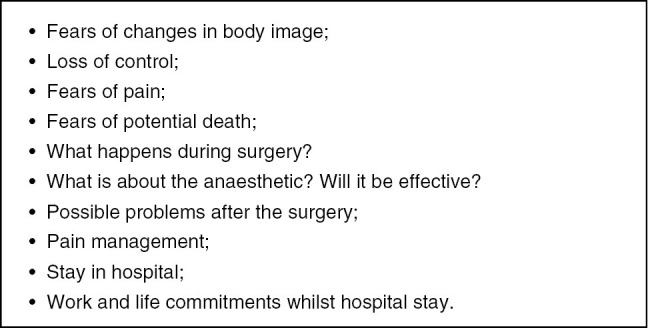
Reasons for perioperative anxiety.

Due to these conditions, there is a need for evidence-based nursing as well as individual support for the treated patient. Otherwise, there is the danger of mechanical nursing, only orienting itself on a medical protocol [14] but without recognizing the individual needs of the patient. Nurses must know the procedures during the entire perioperative process, but they should also notice and look after the individual health situation of the patient and his or her individual needs. Patients do have a gamut of physiological, psychological, and emotional responses to surgery (Figure 1).

**Figure 2:**
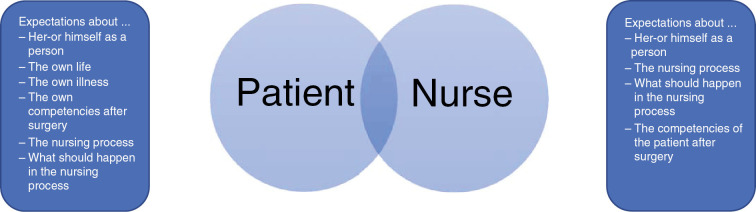
Core of nursing care.

Nurses must recognize this and appropriately consider the nursing process. While there is a nursing routine on one side, there is an exceptional situation for the patient on the other side. Nurses should apply their evidence-based knowledge to each individual case. While the nurse is the expert on the perioperative procedure, the patient is the expert of himself or herself, and both views are equally relevant to the nursing care process. Both the patient and the nurse have their own expectations about nursing care and the caring process itself (Figure 2). The relationship between these two partners is the core of nursing care and determines each nursing interaction.

When nurses pay attention to these necessities, nursing expertise can be a significant and valuable input for a better patient outcome and satisfaction. Besides the more technical tasks, nurses are the ones who communicate and provide information and psychological support. They make the discharge planning regarding the competencies in activities of daily living of the patient and secure the planned discharge of the patient. These duties become more important because of the shortened hospital lengths of stay. Patients need more information and education for the immediate time period after discharge [14], as they cannot single-handedly manage their recovery at home. They usually are insecure, do not act according to the needs of the recovery process, and thus jeopardize the outcome of the surgery.

## Nursing qualification and patient outcomes

Nurses have to handle these complex situations; however, they only are able to do so when they have appropriate nursing expertise in the subject area and are able to apply their general knowledge in the respective situation. Hence, a high-quality nursing education is imperative. Due to the aging of patients, the increase of chronic illnesses, and the shortened hospital length of stay, nursing care has become increasingly complex, which must be countered by a corresponding mix of qualifications.

International results of studies from European countries show a dependence of patient outcomes on the presence of appropriately trained nurses [15]. The mortality rates of patients grow as the number of nursing staff decreases. The presence of a higher number of nursing assistants is also associated with higher mortality. Aiken et al. showed that hospitals with a higher proportion of professional nurses had lower patient mortality. They demonstrated that an increase of 10% in the proportion of professional nurses is associated with a decrease of 11% in patient deaths after general surgery [16]. Further, nurses in a hospital with a higher proportion of professional nurses rate the quality of care in their hospital higher, report better patient safety, and experience lower rates of burnout symptoms. Other studies also found a decrease of adverse patient events such as falls, re-admissions, medication errors, pressure ulcers, or urinary tract infections when a higher proportion of professional nurses were involved [15], [17]. These results show the importance of the presence of professional nurses to care for surgical patients. Regarding the studies listed here, it must be noted that in the countries included in these studies, professional nurses generally hold at least a bachelor’s degree, thus exceeding the conventional education level of nurses in Germany.

The requirements listed above for successful nursing show the complexity of nursing care. There is a clear requirement and demand that Germany also needs a higher proportion of nurses with at least a bachelor’s degree to enable them to deal with the changing requirements in nursing care. We are still at the beginning of the process; thus far, only a few nurses have acquired a bachelor’s degree. Planning for the future, we will need a lot more nurses with an academic degree to be able to successfully handle the complex requirements of surgical patients.

Yet, not only a higher degree is necessary but also the level of staffing must be prioritized. Griffiths et al. [18] have shown that with lower level of staffing, patients have a higher risk of death. Moreover, a reduction of professional nurses to 50% leads to an increase in patient mortality by 21%. Furthermore, patients give a hospital a lower rating when there is a low proportion of professional nurses, independent of the total number of nursing staff. We do not have studies from Germany, but it is to be assumed that we would have similar or worse results. The complexity of the patients’ health situation with a high proportion of chronic illnesses and multimorbidities is the same as in other European countries, but the level of training of German nurses lags behind as well as the number of nursing staff. In Germany, nurses need to care for more patients than in other countries. The nurse/patient ratio is 1:13, while in the United States the ratio is 1:5.3 and in Sweden it is 1:7 [19]. This high number of patients combined with already high numbers of admissions have a negative effect on the quality of nursing care and, thus, on the patient outcomes [18].

## Changes in surgical nursing

The need for higher nursing education to increase nursing competencies, the nurse staff shortage, as well as the health challenges posed by demographic changes all demand an equal change in nursing care. Mitchel has described the necessity of changes in nursing practice as well as in education [14]. He demands the implementation of an extended assessment. When nurses make the first assessment, sometimes as a pre-assessment, they should also note the health situation of the patient and the care requirements to be derived therefrom. Hence, it seems necessary to assess not only physical aspects but also social and psychological information for an appropriate early planning of the necessary perioperative care as well as an early discharge, especially for minimal-stay surgery.

Another aspect is the early information and education of patients to ensure good recovery. When the patient becomes an active and involved partner in his or her care, he or she has the feeling of autonomy and self-determination, which helps with the maintenance or promotion of the patient’s self-management resources. As shown in Figure 2, the patient has his or her own expectations about his or her life and health status, and what will happen in the nursing process. Although these expectations sometimes do not coincide with the assessments of the caregiver, they must be taken into account, just like the patient’s desires and needs, as it is the only way to ensure a rapid and enhanced recovery. Self-management increases with the possibility to actively participate in one’s own therapy [20]. However, to achieve this, patients need information pertaining to their individual pre-existing knowledge as well as their health literacy [20]. Nurses have to recognize the postoperative procedures and the need for support of the patients as well as the need for help after hospital discharge. This is often not a sufficiently considered aspect at present; however, it should be emphasized not only because of the decreasing hospital length of stay but also for nurses who are in dire need of a broad-spectrum evidence-based competence base.

## Conclusions

Nursing plays an important part in the care of surgical patients. Hence, nurses have to take on new tasks due to changing circumstances in the care for inpatients and the changing requirements to their work in a multidisciplinary team. To adapt to these changes, nurses have to increase their competencies, which would result from attaining an appropriate academic degree based on evidence. In addition, there is also a need for another teamwork approach and additional responsibilities among the different health occupational groups. These can be achieved from changes in nursing skills and will contribute to better care.

## Supplementary Material

The article (https://doi.org/10.1515/iss-2019-0010) offers reviewer assessments as supplementary material.
