# Supplementary material for: Nursing care and outcome in surgical patients – why do we have to care?
Source: Innov Surg Sci. 2019 Jul 25;4(4):139–43. doi: 10.1515/iss-2019-0010 (PMC8059353; doi:10.1515/iss-2019-0010)
Supplement: Supplementary file 1 [file iss-04-20190010-s001.pdf]

Reviewer Assessment

Nadja Nestler*

Nursing care and outcome in surgical patients – why do we have to care?

https://doi.org/10.1515/iss-2019-0010
Received May 15, 2019; accepted June 24, 2019

***Corresponding author: Asst. Prof. Dr. Nadja Nestler,** Institute of Nursing Science and Practice, Paracelsus Medical University, Strubergasse 21, 5020 Salzburg, Austria, E-mail: Nadja.nestler@pmu.ac.at. https://orcid.org/0000-0002-7342-0394

Reviewers' Comments to Original Submission

Reviewer 1: Heiner Wenk

Jun 19, 2019

**Reviewer Recommendation Term:**	**Accept**
**Overall Reviewer Manuscript Rating:**	**80**
Is the subject area appropriate for you?	3
Does the title clearly reflect the paper's content?	4
Does the abstract clearly reflect the paper's content?	4
Do the keywords clearly reflect the paper's content?	5 - High/Yes
Does the introduction present the problem clearly?	4
Are the results/conclusions justified?	4
How comprehensive and up-to-date is the subject matter presented?	3
How adequate is the data presentation?	3
Are units and terminology used correctly?	4
Is the number of cases adequate?	N/A
Are the experimental methods/clinical studies adequate?	N/A
Is the length appropriate in relation to the content?	4
Does the reader get new insights from the article?	3
Please rate the practical significance.	3
Please rate the accuracy of methods.	N/A
Please rate the statistical evaluation and quality control.	N/A
Please rate the appropriateness of the figures and tables.	3
Please rate the appropriateness of the references.	5 - High/Yes
Please evaluate the writing style and use of language.	4
Please judge the overall scientific quality of the manuscript.	3
Are you willing to review the revision of this manuscript?	Yes

Comments to Author:

Nurses have an important role in patient care. The article demonstrates the role of education in this field. Change of the Tasks of nurses are explained. Important article.

Open Access. © 2019 Nestler N., published by De Gruyter. This work is licensed under the Creative Commons Attribution 4.0 Public License.

Reviewer 2: Thomas Becker

Apr 14, 2019

**Reviewer Recommendation Term:**	Accept
**Overall Reviewer Manuscript Rating:**	N/A
Is the subject area appropriate for you?	3
Does the title clearly reflect the paper's content?	4
Does the abstract clearly reflect the paper's content?	3
Do the keywords clearly reflect the paper's content?	3
Does the introduction present the problem clearly?	3
Are the results/conclusions justified?	4
How comprehensive and up-to-date is the subject matter presented?	4
How adequate is the data presentation?	3
Are units and terminology used correctly?	4
Is the number of cases adequate?	N/A
Are the experimental methods/clinical studies adequate?	N/A
Is the length appropriate in relation to the content?	4
Does the reader get new insights from the article?	3
Please rate the practical significance.	3
Please rate the accuracy of methods.	N/A
Please rate the statistical evaluation and quality control.	N/A
Please rate the appropriateness of the figures and tables.	3
Please rate the appropriateness of the references.	4
Please evaluate the writing style and use of language.	4
Please judge the overall scientific quality of the manuscript.	3
Are you willing to review the revision of this manuscript?	Yes

Comments to Author:

none
